# Lipid interactions and angle of approach to the HIV-1 viral membrane of broadly neutralizing antibody 10E8: Insights for vaccine and therapeutic design

**DOI:** 10.1371/journal.ppat.1006212

**Published:** 2017-02-22

**Authors:** Adriana Irimia, Andreia M. Serra, Anita Sarkar, Ronald Jacak, Oleksandr Kalyuzhniy, Devin Sok, Karen L. Saye-Francisco, Torben Schiffner, Ryan Tingle, Michael Kubitz, Yumiko Adachi, Robyn L. Stanfield, Marc C. Deller, Dennis R. Burton, William R. Schief, Ian A. Wilson

**Affiliations:** 1 Department of Integrative Structural and Computational Biology, The Scripps Research Institute, La Jolla, California, United States of America; 2 International AIDS Vaccine Initiative (IAVI) Neutralizing Antibody Center, The Scripps Research Institute, La Jolla, California, United States of America; 3 Collaboration for AIDS Vaccine Discovery (CAVD), The Scripps Research Institute, La Jolla, California, United States of America; 4 Center for HIV/AIDS Vaccine Immunology and Immunogen Discovery, The Scripps Research Institute, La Jolla, California, United States of America; 5 Department of Immunology and Microbiology, The Scripps Research Institute, La Jolla, California, United States of America; 6 Applied Physics Laboratory, Johns Hopkins University, Baltimore, Maryland, United States of America; 7 Ragon Institute of MGH, MIT and Harvard, Cambridge, Massachusetts, United States of America; University of Zurich, SWITZERLAND

## Abstract

Among broadly neutralizing antibodies to HIV, 10E8 exhibits greater neutralizing breadth than most. Consequently, this antibody is the focus of prophylactic/therapeutic development. The 10E8 epitope has been identified as the conserved membrane proximal external region (MPER) of gp41 subunit of the envelope (Env) viral glycoprotein and is a major vaccine target. However, the MPER is proximal to the viral membrane and may be laterally inserted into the membrane in the Env prefusion form. Nevertheless, 10E8 has not been reported to have significant lipid-binding reactivity. Here we report x-ray structures of lipid complexes with 10E8 and a scaffolded MPER construct and mutagenesis studies that provide evidence that the 10E8 epitope is composed of both MPER and lipid. 10E8 engages lipids through a specific lipid head group interaction site and a basic and polar surface on the light chain. In the model that we constructed, the MPER would then be essentially perpendicular to the virion membrane during 10E8 neutralization of HIV-1. As the viral membrane likely also plays a role in selecting for the germline antibody as well as size and residue composition of MPER antibody complementarity determining regions, the identification of lipid interaction sites and the MPER orientation with regard to the viral membrane surface during 10E8 engagement can be of great utility for immunogen and therapeutic design.

## Author summary

The trimeric Env glycoprotein located on HIV surface is the target of broadly neutralizing antibodies and is the focus of vaccine and therapeutic approaches to prevent HIV infection. Structural studies with HIV Env trimers have shed light on the complete epitopes of several broadly neutralizing antibodies. However, structural determination of the complete epitopes of the highly cross-reactive MPER antibodies has been technically difficult due to the viral membrane component and that these epitopes are probably only exposed transiently after Env engages CD4. In this study, we structurally characterize the interaction of the broadest and most potent MPER-targeting antibody, 10E8, with viral membrane lipids and scaffolded MPER and propose how 10E8 approaches the MPER-viral membrane epitope during neutralization. Our results indicate that 10E8 interacts with the viral membrane via its light chain and engages MPER in an upright orientation with respect to the HIV-1 membrane. These findings are of interest for design of HIV-1 vaccines and therapeutics.

## Introduction

The HIV-1 envelope protein (Env), a hetero-trimer of non-covalently linked gp120 and gp41 subunits, is the target of broadly neutralizing antibodies (bnAbs) [1]. BnAbs recognize several sites of vulnerability on Env [2]. The membrane proximal external region (MPER) of Env is one of its most conserved regions [3] and, hence, the focus of vaccine and therapeutic design efforts. Previously, several models have been proposed for the MPER orientation with respect to the viral membrane and for the mechanism by which MPER antibodies approach their respective epitopes *in vivo* [4–7]. Several reports suggested that the MPER is relatively inaccessible to antibodies in the pre-fusion conformation and is exposed only transiently after CD4 binding [4,8–10]. Other studies suggested that the MPER is partially laterally inserted into the viral membrane [6] in its pre-fusion form and that neutralization would require antibodies to "extract" the MPER from the membrane [5,7]. Early cryo-electron tomography (cryoET) of native HIV-1 [11] and SIV [12] Env on virions interpreted the MPER and transmembrane (TM) region as three independent helices organized in a tripod-like fashion followed by a turn at Lys683 (the last residue of the gp41 ectodomain; UNIPROT ID: Q70626, HIV1-LW123 numbering). In other cryoET studies [13,14], the gp41 stem was proposed to adopt a compact stalk organization within the trimer suggesting instead an extended, intertwined helical architecture for the MPER and TM region. An extended helical conformation was observed in a recent NMR structure of a construct spanning the MPER and part of the gp41 TM domain that showed no break in helicity at Lys683 [15] and also in the NMR structure of the gp41 TM, which revealed a triple-helix, quaternary TM organization in bicelles [16]. X-ray crystallography and EM [17–19] studies have described in atomic detail the structure of a soluble, stabilized Env construct (BG505 SOSIP.664 gp140 trimer), but these SOSIP structures lack the MPER, TM and cytoplasmic domains. In a recent 4.2 Å resolution cryo-EM structure of a native HIV-1 Env trimer (ΔCT) containing the MPER and TM domains in complex with antibody PGT151, the micelle-embedded TM domain could not be resolved, but the structure also suggested that the MPER may be inaccessible in the pre-fusion form of Env [20]. Furthermore, in the presence of MPER antibody 10E8, an 8.8 Å resolution cryo-EM structure illustrated that the three MPER epitope regions within the trimer form a triple helix [20] and the 10E8-bound Env appears to be elevated off the micelles. This elevation in comparison to the pre-fusion form suggested how the MPER may be engaged by 10E8, but the presence of detergent and the lack of a membrane in that study limited the ability to draw definitive conclusions about 10E8 engagement of native Env on virions. Due to the proximity of MPER to the membrane, MPER binding antibodies are thought to interact with the membrane and, indeed, some have been shown to interact with lipids [21–24].

Several bnAbs target the MPER, with 10E8 and 4E10 neutralizing about 98% of all HIV-1 subtypes tested [25]. 10E8 is the most potent of the MPER antibodies and lacks the polyreactivity of 4E10. 10E8 and 4E10 target the same helical epitope (C-terminal MPER residues 671–683) but differ in their modes of binding [25–27]. Residues 672–674 adopt a 3_10_ helical turn when engaged by 4E10, but are part of a continuous α helix (region 671–683) when bound by 10E8. The 2F5 epitope (residues 656–671) [28,29], which is N-terminal to the 10E8 and 4E10 epitopes, adopts an extended conformation with a type 1 β-turn and the Z13e1 epitope (residue 668–677) [30] consists of linked helical turns. Although these antibodies target linear MPER epitopes on gp41 [25,26,29,30], binding to individual lipids [21] or liposomes [31] also suggests that their complete epitopes include viral membrane components. Indeed, studies of MPER antibodies suggest a role for their complementarity determining region (CDR) H3 in binding to viral membrane [22,24,31–34], but no clear picture has emerged as to how each antibody is oriented with respect to the MPER and viral membrane during engagement. To determine which regions of 10E8 might interact with the HIV-1 membrane, and motivated by our recent findings regarding 4E10 interaction with lipids that compose the viral membrane [23], we undertook a crystallographic study of 10E8 in complex with lipids and an epitope scaffold [35]. We complemented our observations of 10E8-lipid binding with binding and neutralization experiments as well as structure determination of several 10E8 mutants. Our combined results have identified which 10E8 regions interact with the viral membrane and indicated that the MPER likely adopts an upright orientation with respect to the viral membrane during antibody engagement, as we also proposed for 4E10 [23]. Our high-resolution structural study increases our understanding of the relative MPER location, orientation and conformation during MPER antibody binding, and provides insights for the design of immunogens and therapeutic antibodies.

## Results

To aid in characterization of the complete 10E8 epitope consisting of MPER and lipids, we designed an epitope-scaffold that presented the MPER on a stable framework in the 10E8-bound conformation derived from previous crystal structures [25,27]. T117v2 is a variant of the previously reported T117 epitope-scaffold [35] that presents the 10E8 core helical epitope residues and binds to mature 10E8 with 29 pM affinity (Table 1). Whereas T117 was originally designed as an epitope-scaffold for 4E10, T117v2 was modified by M125K and D126A mutations to accommodate binding by 10E8. The crystal structure of this scaffold in complex with 10E8 was solved at 2.1 Å resolution and revealed similar conformations for the antibody and MPER epitope (S1 Fig) as in the 10E8-peptide structure (PDB 4G6F [25]) to which it aligns with a Cα r.m.s.d. of 0.31 Å calculated from the Fab variable regions and epitope residues of the respective structures.

**Table 1:** 10E8 variant binding to the T117v2 scaffold.

Analyte	Mutations on light-chain surface	T117v2		
		k_on_ (1/Ms)	k_off_ (1/s)	K_D_ (M)
10E8 IgG mature	N/A	3.17E+06	9.04E-05	2.85E-11
10E8 IgG mutant 1	Arginines reverted to germline residues (R17Q^(L)^, R24Q^(L)^, R70T^(L)^);	3.57E+06	4.00E-05	1.12E-11
10E8 IgG mutant 2	Arginines replaced with aspartate or glutamate (R17D^(L)^, R24E^(L)^, R29E^(L)^, R70E^(L)^)	2.80E+06	4.60E-05	1.64E-11
10E8 IgG mutant 3	Basic residues and polar residues mutated to aspartate or glutamate (R17D^(L)^, R24E^(L)^, R29E^(L)^, K51D^(L)^, N52D^(L)^, S65D^(L)^, S67D^(L)^, R70E^(L)^, S76D^(L)^)	5.59E+05	2.11E-04	3.79E-10
10E8 IgG mutant 4	Residues flanking lipid head group mutated to glutamate (R29E^(L)^ and Y32E^(L)^)	1.08E+06	1.27E-03	1.17E-09
10E8 IgG mutant 5	Residues flanking lipid head group mutated to alanine (R29A^(L)^ and Y32A^(L)^)	1.30E+06	1.60E-04	1.23E-10

### Identification of 10E8 regions interacting with the viral membrane

**Fig 1:**
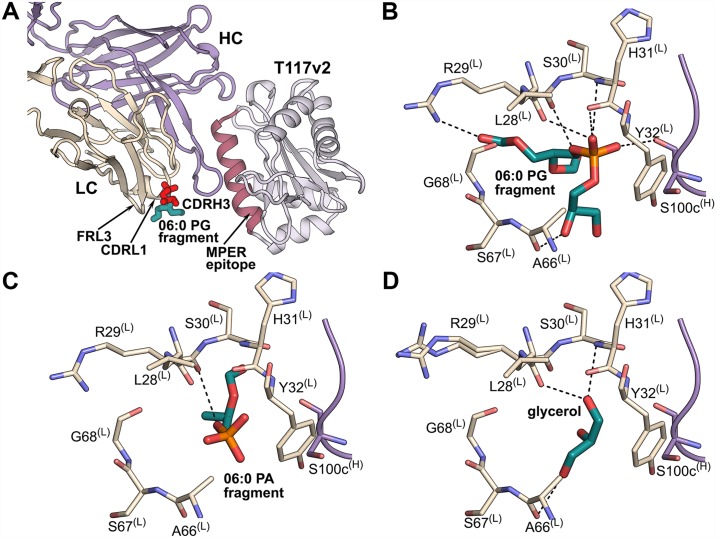
10E8 lipid-binding site. (A) Crystal structure of the 10E8-T117v2 complex bound to 06:0 PG (sticks; red, glycerol and phosphate moieties of the head group; cyan, head group region linked to the lipid tails). The 10E8 light chain (LC) is shown in beige, heavy chain (HC) in violet, and T117v2 scaffold containing the MPER epitope (pink) in gray. This color scheme is used throughout. (B) All potential hydrogen-bond interactions (within ~3.5 Å) of the 06:0 PG fragment (colored by atoms) with 10E8 residues of CDRL1 (beige) and CDRH3 (violet) are shown as dashed lines. (C) All potential hydrogen-bond interactions (within ~3.5 Å) of the 06:0 PA fragment (colored by atoms) with CDRL1 (beige) and CDRH3 (violet) residues of 10E8. (D) All potential hydrogen-bond interactions (within ~3.5 Å) of a glycerol found in the lipid-binding site in the 10E8-T117v2 structure in the absence of added lipids.

Crystal structures of 10E8 in complex with T117v2 epitope scaffold and lipids 06:0 phosphatidylglycerol (PG) (2.37 Å resolution) or 06:0 phosphatidic acid (PA) (2.62 Å resolution) led to identification of a lipid-binding site at the proximity of the CDRL1 and CDRH3 loops (Fig 1A). Electron density for the lipid head groups and part of the PG acyl tail were observed (S2 Fig). The orientation of the lipid fragments with respect to 10E8 suggests that the hydrophobic lipid tails do not interact with the Fab or T117v2 and are disordered. The 06:0 PG and 06:0 PA head groups are bound into a crevice delineated by CDRL1 (Leu28^(L)^, Arg29^(L)^, Ser30^(L)^, His31^(L)^ and Tyr32^(L)^), FRL3 (Ala66^(L)^, Ser67^(L)^ and Gly68^(L)^), and by Trp100b^(H)^, Ser100c^(H)^ and Gly100d^(H)^ at the CDRH3 tip (Fig 1B and 1C; where ^(L)^ stand for light and ^(H)^ for heavy chains). Glycerol, the cryo-protection component of the crystals, occupies the lipid-binding site in the 10E8-T117v2 structure when exogenous lipids are not added in the crystallization experiments (Fig 1D).

**Fig 2:**
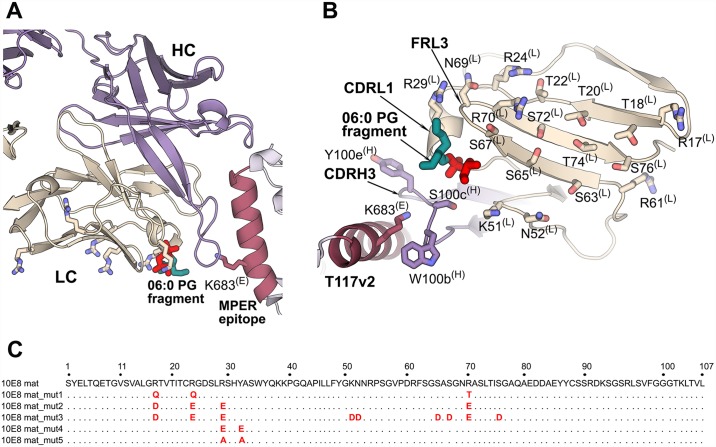
Design of 10E8 light-chain mutants. (A) Several light chain (LC; beige) basic residues (Arg and Lys shown as sticks) are in the same plane with the lipid head group (red and cyan sticks) and Lys683 (red sticks) of the gp41 MPER epitope (red). The heavy chain (HC) is shown in violet. (B) Light-chain surface residues (beige) that are presumed to face the viral membrane. The heavy-chain residues at the tip of CDRH3 are shown as violet sticks. The 10E8 peptide epitope (pink) from the T117v2 scaffold is shown up to Lys683 (pink; last residue of gp41 ectodomain). (C) Alignment of the amino-acid sequence of the five 10E8 light-chain mutants compared to wild-type 10E8. Only the variable region of the 10E8 light-chain variants are shown with the Kabat numbering scheme on top of the alignment. The mutations are highlighted in red.

Interestingly, the 10E8 light chain displays basic surface patches arising from somatically mutated (Arg17^(L)^, Arg24^(L)^, Arg70^(L)^), and germline-encoded (Arg29^(L)^, Lys51^(L)^, Arg61^(L)^) residues in a plane with the bound lipid head groups observed in our structures, as well as with K/R683, the last residue of the gp41 ectodomain before the TM region (Fig 2A). Thus, this basic as well as Ser/Thr/Asn rich polar surface (Fig 2B) is likely to interact with polar and negatively charged lipid head groups of the viral membrane [36].

### Design of light-chain mutants to probe the orientation of 10E8 with respect to the membrane

To further investigate and validate that the light chain of 10E8 is involved in binding to the lipid head groups of the membrane upper leaflet, we designed several 10E8 light-chain mutants (Fig 2C): mutant 1 reverts the three somatically mutated arginines to 10E8 germline residues (R17Q^(L)^, R24Q^(L)^, R70T^(L)^); mutant 2 replaces most of the lipid-proximal arginine residues with aspartate or glutamate (R24E^(L)^, R29E^(L)^, R70E^(L)^, as well as R17D^(L)^); mutant 3 has all the basic residues as well as some of the polar residues on this surface mutated to D or E (R17D^(L)^, R24E^(L)^, R29E^(L)^, K51D^(L)^, N52D^(L)^, S65D^(L)^, S67D^(L)^, R70E^(L)^, S76D^(L)^); mutant 4 has R29^(L)^ and Y32^(L)^, which flank the lipid head groups, mutated to glutamate (R29E^(L)^ and Y32E^(L)^); and mutant 5 has R29^(L)^ and Y32^(L)^ mutated to alanine (R29A^(L)^ and Y32A^(L)^). Overall, mutants 1 to 3 were constructed to coat the presumed membrane-interacting surface of 10E8 with different amounts of negative charge to investigate binding affinity and specificity of the 10E8 epitope (protein and lipid) and to examine the effects of the mutations on neutralization potency. In addition, mutants 4 and 5 were designed to disrupt the lipid-binding site that we observe in the crystal structures.

### Influence of light-chain mutations on binding to the epitope scaffolds

To investigate the effect of the light-chain surface mutations on 10E8 IgG binding to the T117v2 scaffold, we performed surface plasmon resonance (SPR) experiments. SPR analysis (Table 1 and S3 Fig) shows that, with the exception of mutant 4 for which the residues involved in lipid head group binding were mutated to glutamate (R29E^(L)^ and Y32E^(L)^), all other mutants retained picomolar affinity (K_D_) to the T117v2 scaffold (Table 1). Mutations R29E^(L)^ and Y32E^(L)^ in mutant 4 (1.2 nM K_D_ for T117v2) resulted in a ~ 40 fold reduction in binding compared to the affinity-matured 10E8 IgG (0.029 nM for T117v2), while mutation of the same residues to alanine in mutant 5 (~0.12 nM K_D_) resulted in only a 4-fold decrease in binding. As 10E8 interaction with the MPER peptide epitope is only with the heavy chain (Fig 1A), these results indicate that, except for mutant 4, all other light-chain mutants do not interfere with 10E8 binding to the MPER-scaffold.

Structure determination and neutralization potency experiments with the best binding mutants were then performed to determine if changing the light-chain surface charge by mutation leads to conformational changes or compromises neutralization. Abolition or decrease in neutralization would suggest that the 10E8 light chain is oriented toward and interact with the viral membrane. We focused on mutants 1–3 and 5, as binding to the peptide-scaffolded T117v2 was decreased for mutant 4.

### Structural characterization of the 10E8 LC mutants

**Fig 3:**
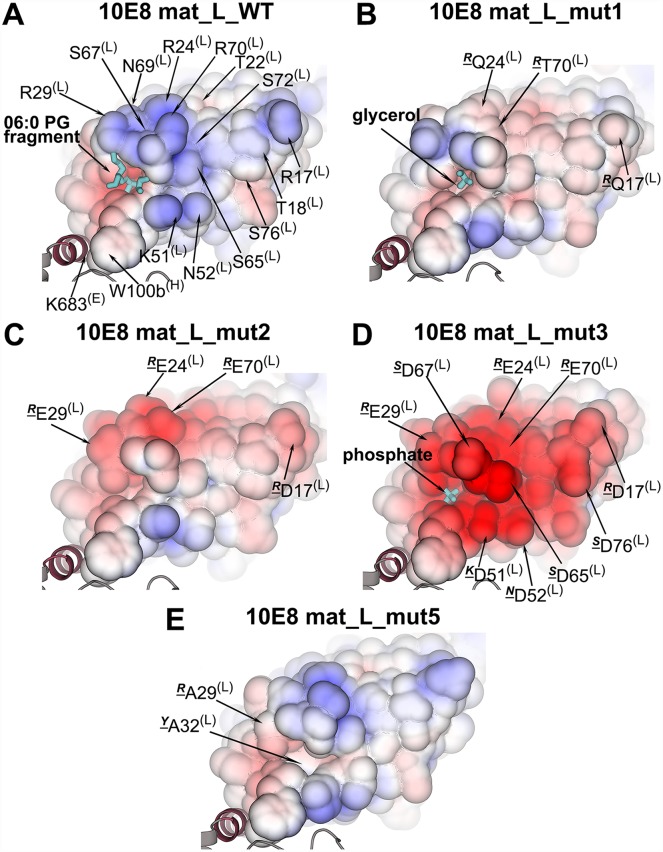
Distribution of charge on the surfaces of the 10E8 light-chain variants. The electrostatic solvent accessible surface (contoured at ±5 kT/e) of the light-chain region presumed to bind the viral membrane is shown for: (A) wild-type mature 10E8; (B) 10E8 mutant 1; (C) 10E8 mutant 2; (D) 10E8 mutant 3; (E) 10E8 mutant 5. Negative and positive charges are indicated in red and blue colored surfaces, respectively. The ligands bound in the lipid-binding site are shown as dark cyan sticks and the epitope scaffold is shown in gray. The underlined superscript letters designate the original residues in the 10E8 wild type.

X-ray structures of mutants 1–3 and 5 (Table 1) were determined in complex with the T117v2 scaffold at 1.6 Å (mutant 1) and 2.0–2.2 Å (mutants 2, 3 and 5) resolution. Superposition of the Cα atoms of the variable domains of mutants and wild-type 10E8 shows nearly identical conformations (Cα r.m.s.d. of 0.40, 0.23, 0.31 and 0.39 Å for mutants 1, 2, 3, and 5, respectively). Thus, the mutations mainly result in different charge distributions on the light-chain surface (Fig 3A–3E), with the most negative surface observed for mutant 3. Although mutant 1 has a slightly larger r.m.s.d. (0.4 Å), the differences in the main chain occur mainly at the Fab elbow region. Only in mutant 5 do the R29A^(L)^ and Y32A^(L)^ mutations produce a slight shift in the main-chain for FRL2 (residues 48, 49), CDRL2 (50–56), FRL3 (57–72) and CDRL1 (24–32) with maximum Cα differences observed in CDRL2 (1.3 Å for Lys51^(L)^), FRL3 (0.9 Å for Gly68^(L)^) and CDRL1 (0.9 Å between Arg29^(L)^ and Ala29^(L)^; S4 Fig). The shift for CDRL2 in mutant 5 may be the direct result of substituting Tyr32^(L)^ with alanine, which allows the nearby Phe48^(L)^ side chain to rotate and influence the conformation of Lys51^(L)^, Asn52^(L)^ and Asn53^(L)^. None of the mutations affect binding to T117v2 (in agreement with the SPR study, Table 1) as the mutant CDRH3 conformations are almost identical to wild-type 10E8 (S4 Fig).

The four mutant structures show different bound ligands (Fig 3B–3E) depending on the residue mutated and on the components of the crystallization mother liquor and cryoprotectants (n.b. all mutants were crystallized without lipids and in different conditions). Mutant 1 has glycerol from the cryoprotectant in the lipid-binding site (Fig 3B; S2 Table; S5A–S5C Fig), as also observed for the wild-type 10E8-T117v2 complex cryoprotected with glycerol (Fig 1D; S2E and S2F Fig). In mutant 2 for which Arg29^(L)^ was mutated to glutamate, only water molecules seem to occupy this site when ethylene glycol was used for cryoprotection rather than glycerol. However, in mutant 3, with the same mutation (R29E^(L)^), a phosphate from the crystallization condition occupies the site (S5E and S5F Fig) suggesting that replacement of Arg29^(L)^ with a negative charge might still retain binding to at least a phosphate, which is a component of phospholipid head groups (n.b. glycerol was absent from the buffers used with crystallization of mutant 3, and thus does not compete for phosphate binding to this site). In mutant 5, where R29^(L)^ and Y32 ^(L)^ are mutated to alanine (Fig 3E), glycerol (used for cryoprotection) is not observed at this location in either of the two Fabs in the asymmetric unit, suggesting that the lipid binding site is severely disrupted by these mutations.

### Neutralization potency of the 10E8 light-chain mutants

In parallel, we probed the neutralization potency of 10E8 mutants 1–3 and 5 against a panel of 109 viruses [37] to determine if these mutations in the light chain interfere with neutralization. A decrease in neutralization potency compared to wild type was observed with most of the strains for mutant 1 in which three arginines (two are proximal to the lipid-binding site) were reverted to the germline residues (Fig 3B and Table 2). A more significant decrease in neutralization was observed for mutant 2 (Fig 3C and Table 2). Increasing the amount of negatively charged residues on the light-chain surface in mutant 3 results in loss of neutralization (Fig 3D and Table 2) of most of the viruses that we tested with the breadth dropping to 34%. Interestingly, mutation of only R29^(L)^ and Y32^(L)^ to alanine in mutant 5, which was designed to disrupt the observed lipid-binding site (Fig 3E), also abrogated the neutralization of most of the viruses tested (breadth of 44%, Table 2), despite retaining picomolar binding affinity to the epitope scaffolds. Thus, mutation of basic and polar residues on the light-chain surface to acidic residues does not interfere with binding to the MPER peptide epitope, but neutralization potency and breadth decreased as the number of negatively charged residues on the surface increased. Substantial loss of neutralization occurred when the lipid-binding site was disrupted (mutant 5) or when the light-chain surface patch contained mainly acidic residues (mutant 3).

**Table 2:** Neutralization breadth and potency of wild-type 10E8 and 10E8 light-chain mutants assayed on an indicator panel of hundred nine pseudotype viruses [37].

virus	clade	10E8 WT	10E8 mut 1	10E8 mut 2	10E8 mut 3	10E8 mut 5	virus	clade	10E8 WT	10E8 mut 1	10E8 mut 2	10E8 mut 3	10E8 mut 5
6535.3	B	0.013	0.063	0.660	> 50	> 50	CNE53	BC	0.004	0.171	1.53	23.1	10.6
QH0692.42	B	0.023	0.502	2.646	29.8	9.31	CNE58	BC	0.008	0.089	0.952	20.1	12.3
SC422661.8	B	0.011	0.143	0.954	> 50	10.3	MS208.A1	A	0.240	0.035	11.2	> 50	> 50
PVO.4	B	0.123	1.339	15.5	> 50	> 50	Q23.17	A	0.093	0.250	2.57	> 50	> 50
TRO.11	B	0.000	0.005	0.164	32.3	> 50	Q461.e2	A	0.144	1.09	17.9	> 50	> 50
AC10.0.29	B	0.019	0.502	5.12	> 50	18.1	Q769.d22	A	0.063	0.641	13.2	> 50	> 50
RHPA4259.7	B	0.016	0.342	7.24	> 50	> 50	0260.v5.c36	A	0.701	6.73	> 50	> 50	> 50
REJO4541.67	B	0.005	0.116	0.126	30.7	7.36	191955_A11	A (T/F)	0.165	0.864	9.02	> 50	> 50
TRJO4551.58	B	0.145	0.853	9.09	> 50	> 50	191084_B7-19	A (T/F)	0.082	2.32	33.2	> 50	> 50
WITO4160.33	B	0.015	0.277	10.2	> 50	> 50	T257-31	CRF02_AG	0.121	0.912	2.89	> 50	50.0
CAAN5342.A2	B	0.040	1.64	13.0	> 50	> 50	928–28	CRF02_AG	0.008	0.067	0.339	19.4	6.15
WEAU_d15_410_5017	B (T/F)	14.7	> 50	> 50	> 50	> 50	263–8	CRF02_AG	0.016	0.003	0.049	11.7	6.24
1006_11_C3_1601	B (T/F)	0.076	1.27	8.7	> 50	> 50	T250-4	CRF02_AG	0.282	0.372	4.44	> 50	> 50
1054_07_TC4_1499	B (T/F)	0.000	0.006	0.111	4.94	1.37	T251-18	CRF02_AG	0.027	0.259	1.81	> 50	> 50
1056_10_TA11_1826	B (T/F)	0.002	0.020	0.542	15.2	5.94	T278-50	CRF02_AG	0.025	0.250	2.38	> 50	> 50
1012_11_TC21_3257	B (T/F)	0.052	0.692	5.82	> 50	36.4	T255-34	CRF02_AG	0.083	0.089	5.92	> 50	> 50
6240_08_TA5_4622	B (T/F)	0.106	0.424	3.79	46.4	27.5	211–9	CRF02_AG	0.002	> 50	> 50	> 50	> 50
62357_14_D3_4589	B (T/F)	0.072	0.579	9.87	> 50	> 50	235–47	CRF02_AG	0.041	0.004	0.542	> 50	33.7
SC05_8C11_2344	B (T/F)	0.078	0.080	0.471	21.0	2.28	620345.c01	CRF01_AE	7.69	0.032	5.18	47.2	> 50
Du156.12	C	0.005	0.001	0.017	1.1	0.685	C1080.c03	CRF01_AE	0.043	0.019	0.174	4.49	1.20
Du172.17	C	0.005	0.086	0.566	16.3	7.60	R2184.c04	CRF01_AE	0.128	0.071	1.43	21.1	25.3
Du422.1	C	0.009	0.089	0.508	9.08	3.02	R1166.c01	CRF01_AE	0.044	0.103	1.23	8.25	6.97
ZM197M.PB7	C	0.002	0.057	0.624	24.3	7.81	R3265.c06	CRF01_AE	0.472	1.75	20.7	> 50	> 50
ZM214M.PL15	C	0.001	0.870	15.0	> 50	> 50	C3347.c11	CRF01_AE	0.000	0.003	0.001	2.24	1.28
ZM233M.PB6	C	0.049	0.099	0.371	16.0	10.2	C4118.c09	CRF01_AE	0.019	0.846	7.35	> 50	> 50
ZM249M.PL1	C	0.055	0.628	18.4	> 50	> 50	CNE8	CRF01_AE	0.012	0.291	2.11	38.5	22.6
ZM53M.PB12	C	1.51	7.67	> 50	> 50	> 50	CNE5	CRF01_AE	0.063	1.02	13.9	> 50	> 50
ZM109F.PB4	C	0.002	0.219	1.36	26.9	13.5	BJOX009000.02.4	CRF01_AE	0.055	1.15	4.96	> 50	19.2
ZM135M.PL10a	C	0.018	0.147	2.50	24.9	8.76	BJOX015000.11.5	CRF01_AE (T/F)	0.027	0.413	2.62	> 50	21.8
CAP45.2.00.G3	C	0.020	0.809	9.76	> 50	> 50	BJOX010000.06.2	CRF01_AE (T/F)	0.019	0.059	0.188	> 50	2.97
CAP210.2.00.E8	C	0.001	0.278	6.52	> 50	> 50	BJOX025000.01.1	CRF01_AE (T/F)	0.004	0.070	0.925	22.1	2.55
HIV-001428-2.42	C	0.174	0.261	7.46	> 50	> 50	BJOX028000.10.3	CRF01_AE (T/F)	0.001	0.178	1.14	> 50	> 50
HIV-0013095-2.11	C	0.012	0.238	2.08	> 50	15.7	X1193_c1	G	0.001	0.004	0.838	> 50	28.2
HIV-16055-2.3	C	0.009	0.962	7.93	> 50	> 50	P0402_c2_11	G	0.070	0.806	14.3	> 50	> 50
HIV-16845-2.22	C	0.153	0.087	0.635	16.3	10.2	X1254_c3	G	0.536	8.41	> 50	> 50	> 50
Ce0393_C3	C (T/F)	0.002	0.514	5.95	42.0	44.3	X2088_c9	G	> 50	> 50	> 50	> 50	> 50
Ce1176_A3	C (T/F)	0.032	0.084	0.495	14.9	9.37	X2131_C1_B5	G	0.001	0.158	0.131	21.6	3.24
Ce2010_F5	C (T/F)	0.040	0.146	3.29	> 50	> 50	P1981_C5_3	G	0.002	0.005	0.061	2.67	1.47
Ce0682_E4	C (T/F)	0.141	> 50	> 50	> 50	> 50	X1632_S2_B10	G	0.171	0.558	3.46	> 50	50.0
Ce1172_H1	C (T/F)	0.171	1.18	24.6	> 50	> 50	3016.v5.c45	D	0.029	0.080	0.653	14.8	3.48
Ce2060_G9	C (T/F)	0.001	0.005	0.023	5.23	1.06	A07412M1.vrc12	D	0.051	0.428	2.99	> 50	28.5
Ce703010054_2A2	C (T/F)	0.684	4.50	23.6	> 50	> 50	231965.c01	D	0.640	2.87	26.1	> 50	> 50
BF1266.431a	C (T/F)	0.056	0.019	0.781	> 50	> 50	3817.v2.c59	CD	0.016	0.170	1.98	37.5	> 50
246F_C1G	C (T/F)	0.817	3.83	29.8	> 50	> 50	6480.v4.c25	CD	0.704	0.981	10.2	> 50	> 50
249M_B10	C (T/F)	0.022	2.56	38.8	> 50	> 50	6952.v1.c20	CD	0.007	0.085	0.730	8.09	3.39
ZM247v1(Rev-)	C (T/F)	0.032	0.109	0.716	> 50	> 50	6811.v7.c18	CD	0.455	5.14	55.0	> 50	> 50
7030102001E5(Rev-)	C (T/F)	0.305	5.33	> 50	> 50	> 50	89-F1_2_25	CD	0.305	3.32	23.2	> 50	67.7
1394C9G1(Rev-)	C (T/F)	0.071	1.15	8.33	> 50	> 50	3301.v1.c24	AC	0.686	4.94	37.7	> 50	> 50
Ce704809221_1B3	C (T/F)	0.001	0.027	0.280	> 50	> 50	6041.v3.c23	AC	0.141	0.609	4.33	> 50	> 50
CNE19	BC	0.148	0.036	1.48	29.9	29.8	6540.v4.c1	AC	0.032	0.289	6.00	> 50	> 50
CNE20	BC	0.024	0.001	0.004	11.8	2.58	6545.v4.c1	AC	0.065	0.252	2.26	> 50	> 50
CNE21	BC	0.093	0.701	7.16	> 50	> 50	0815.v3.c3	ACD	0.000	0.000	2.16	> 50	> 50
CNE17	BC	0.011	0.033	0.012	8.98	11.1	3103.v3.c10	ACD	> 50	> 50	> 50	> 50	> 50
CNE30	BC	0.047	0.120	1.56	> 50	> 50		**Breadth**	**98%**	**95%**	**92%**	**34%**	**44%**
CNE52	BC	0.042	0.001	0.205	> 50	> 50		**Median IC50**	**0.040**	**0.252**	**2.569**	**19.410**	**9.365**

The values are neutralization IC_50_ in μg/ml.

We noticed that our 10E8 wild-type IgG shows about ten-fold higher neutralization potency compared with results reported previously [25]. To probe the quality of our 10E8 and mutants IgG preparations, we performed size exclusion chromatography-multi-angle light scattering (SECMALS) analysis of the samples. 10E8 and mutant 1 showed the presence of three major peaks in each sample. Each of these peaks had a molecular weight of approximately 150 kDa, consistent with the expected size of monomeric antibody (S6 Fig). We attribute this behavior to a slow conformational isomeration of 10E8, which results in differential interaction with the size exclusion matrix as previously reported for a solubility-optimized 10E8 mutant (10E8v4) [38]. Thus, our results suggest conformational isomerization for both 10E8 wild type and mutant 1. In contrast, mutants 2, 3 and 5 each eluted as a single peak with a molecular weight of approximately 150 kDa. The SECMAL analysis shows that the IgG samples used for neutralization are all monomeric with only insignificant amounts (<0.5%) of aggregates observed for wild-type 10E8 and mutant 1 (S6 Fig).

### Structural model of 10E8 binding to the gp41-viral membrane epitope

**Fig 4:**
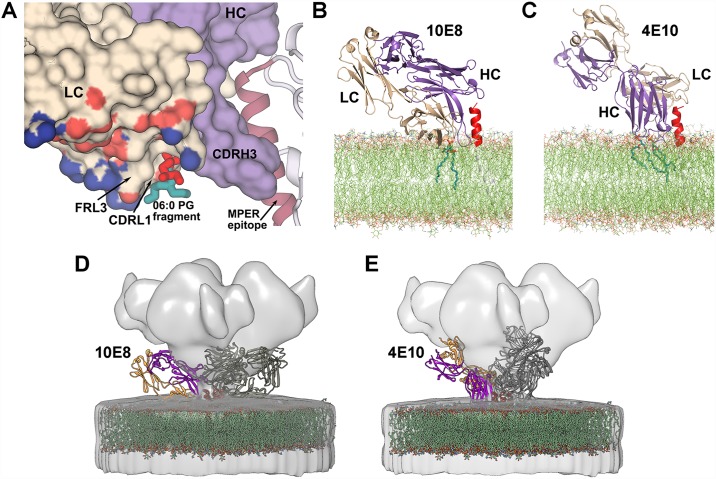
Integration of experimental data into a 10E8-MPER epitope-viral membrane model. (A) Surface rendering of the 10E8 Fab (beige, light chain; violet, heavy chain) showing the PG lipid fragment (red-dark cyan sticks) bound in a cavity formed between CDRL1, FRL3 and CDRH3 in the 10E8-T117v2-PG crystal structure. The helical 10E8 epitope residues in the T117v2 scaffold protein (gray) are shown in dark pink and the light-chain surface interacting with the viral membrane is colored by atom type. (B) Model of the 10E8 angle of approach with respect to MPER epitope-viral membrane during antibody engagement. (C) Model of the 4E10 angle of approach with respect to MPER epitope-viral membrane [23] for comparison. In both (B) and (C), the light and heavy chains are shown in beige and violet, respectively. The helical MPER epitope is shown in red and the lipid bilayer as thin green lines. The experimental bound lipids are shown as thicker dark cyan sticks in both structures. (D) and (E) Fitting our models of the viral membrane with 4E10 and 10E8 in the experimental EM map of the CD4-bound Env (EMDB-5455 [40]). The light and heavy chains are shown as yellow and blue cartoons. The lipid bilayers are shown in green and the MPER epitope as dark red helices.

The structural, neutralization and binding analyses of wild-type and light-chain 10E8 mutants suggest that the 10E8 variable light-chain residues facilitate approach to and interaction with the viral membrane. To obtain further insights into 10E8 binding to gp41 of HIV Env on the viral surface, and encompassing our recent findings on 4E10 binding to the lipids [23], we generated a model of 10E8 bound to the viral membrane-gp41 epitope assembly based on the orientation of the lipids and gp41 observed in our crystal structures (Fig 4A) using the CHARMM force field membrane builder [39]. The viral membrane head groups of the outer leaflet were roughly placed in a plane containing the basic side chains of the 10E8 light-chain, Arg17^(L)^, Arg24^(L)^, Arg29^(L)^ and Arg70^(L)^, Lys51^(L)^, the lipid head group, and gp41 Lys/Arg683 (Fig 4A and 4B). Our model suggests that 10E8 epitope includes the gp41 helical peptide (residues 671–683) tilted about 75–80° from the viral membrane surface and lipid head groups of the membrane. The 10E8 light chain would then face the membrane, with which it interacts via CDRH3, CDRL1, and FRL3, and possibly additional residues (Ser/Thr/Arg/Lys) of the light-chain surface (Fig 4B). Indeed, the relatively large percentage of short polar residues, serine and threonine, on this light-chain β-sheet surface form a flat polar region that can perhaps also interact with the head groups of the various lipids composing the viral membrane. Fitting of the model into the experimental EM map of the CD4-bound Env (EMDB-5455 [40]) shows that 10E8 CDRs H1 and H2 might interact with additional regions of Env (Fig 4D), as also suggested by the cryo-EM structure of 10E8 in complex with the native HIV-1 Env trimer (ΔCT) and PGT151 [20].

## Discussion

The MPER region of gp41, although highly conserved and targeted by several very broadly neutralizing antibodies (10E8, 4E10 and 2F5), has so far not led to a vaccine immunogen that elicits such antibodies. 4E10 and 2F5 antibodies have been studied in great detail [21,26,27,29]. Their binding to phospholipids prompted the suggestion that such antibodies are rarely produced due to tolerance mechanisms resulting from interaction with ‘self-components’ [41–44]. However, the binding of 4E10 to cardiolipin [44] and its overall reactivity profile appears to differ from those of autoimmune antibodies [22,45]. 10E8, which binds to the same MPER epitope region as 4E10, does not show cross-reactivity with cardiolipin or other autoantigens [25], although binding to cholesterol-rich liposomes was recently demonstrated [31].

Our crystallographic study of 10E8 binding to two phospholipids PG and PA reveals a lipid-binding site in a cavity delineated by CDRL1, CDRH3 and FRL3 (Figs 1A and 4A). In healthy cells, PG and PA are less abundant lipids of the plasma membrane [46]. However, the amount of these lipids increases on the HIV-1 membrane that is acquired from the host cell during budding [36,47–49]. A diversity of ligands (PG, PA, phosphate, glycerol) is observed in our structures in the lipid-binding site and, therefore, other phospholipid head groups could potentially occupy this cleft. The lipid binding site residues are relatively conserved in the 10E8 germline IGLV3-19*01 sequence with only two differences observed for light-chain residues 31 (tyrosine in the germline-encoded sequence and histidine in mature 10E8) and 66 (serine in the germline and alanine in mature 10E8). The presence of the tyrosine and serine at the respective positions in germline does not influence the conformation of CDRL1 and FRL3 in this cavity as comparison with the structure of the unbound germline 10E8 (PDB 5JO5 [50]) shows that the side chains of residues 31 and 66 point away from this cleft and do not change the topology of the lipid-binding site. However, CDRH3, which also delineates this cavity, is not visible in the germline structure as its residues appear to be disordered. Thus, it is not clear if the lipid-binding site is fully recapitulated in the 10E8 germline. In addition to the lipid-binding site, we observed that the 10E8 light-chain surface is coated by several basic (arginine and lysine) and multiple polar (serine and threonine) residues, which together with Lys683, the last residue of the gp41 ectodomain, lie in a plane that roughly coincides with the lipid head groups observed in our structure. Several light-chain mutants show similar binding affinity to the epitope scaffold but with decreased neutralization potency and breadth suggesting that, although binding to the MPER peptide epitope is not compromised, binding to its composite epitope formed by MPER peptide and viral membrane lipids on the virus surface would be affected. Furthermore, the light-chain mutations do not alter the Fab conformation, consistent with the binding experiments, but result in more acidic surface, strongly suggesting that the 10E8 antibody interacts with the viral membrane via this basic-polar light-chain patch. Our data also suggest an upright orientation of the MPER helical epitope of 10E8 with respect to the viral membrane (Fig 4B), which is tilted about 78°±3° from the bilayer during antibody engagement (similar to that observed for 4E10 [23]). This angle is defined by the epitope’s helical axis intersecting the axis of the plane of the lipid head group in the direction from which 10E8 approaches the MPER in the CHARM model. This MPER orientation with respect to the membrane and the angle of antibody approach is also suggested by the recent cryo-EM structure of native JR-FL EnvΔCT at 8.8 Å resolution that contains the MPER and TM domains in complex with 10E8 and PGT151 antibodies [20]. This cryo-EM structure provided fascinating new insights into the 10E8 interaction with Env and on new features outside the MPER that are part of the 10E8 epitope (e.g. N88 and N625 glycans), although no detailed information was possible for the 10E8 interaction with membrane lipids (or the micelle in the EM study) or for the TM region. Our study suggests that 10E8 interacts with the helical MPER with an angle of approach of ~43°±3° as measured from the viral membrane to the pseudo dyad axis between the variable light and heavy chains (defined by atoms Cβ of Ser179 and Cα of Phe100a, with the light-chain variable region interacting with the phospholipid head groups on the membrane surface. In this model, CDRH3 inserts between the MPER epitope and the lipid head groups with the aromatic residues at its tip located within the hydrophilic region of the lipid bilayer. It is likely that CDRH1, CDRH2 and FRH3 of 10E8 form additional contacts with Env as shown in our MD model fitted into the EM map of the full-length Env bound to CD4 [40] (Fig 4D) and as shown in the JR-FL EnvΔCT-10E8-PGT151 EM structure [20]. In the JR-FL EnvΔCT-10E8-PGT151 EM structure, the presence of PGT151, which binds with a stoichiometry of two Fabs per trimer, leads to a disruption in the symmetry of the spike causing the three 10E8 Fabs to have slightly different orientations compared to each other (i.e. slightly different angle of tilt toward the micelle position and rotations around the dyad axis between the heavy and light chains). In our model, the three 10E8 Fabs bind with the same angle of approach to their respective MPER-viral membrane composite epitope. The 10E8 Fab in the EM structure with the closest orientation to the one that we propose here is tilted only ~8° more towards the predicted position of the membrane surface. Gp120 conformational changes on receptor and co-receptor engagement could also promote 10E8 binding. Comparison of this 10E8 model with a previous model of 4E10 bound to the gp41 epitope-lipid bilayer (Fig 4B and 4C; [23]) shows a similar orientation of the MPER with regard to the viral membrane. 10E8 and 4E10 both interact with the residues on the same face of the helical MPER epitope, but the Fab variable regions that contact the gp41 epitope differ. The two antibodies are rotated by about 90° around their pseudo-dyad axes with respect to each other, with 10E8 interacting with the viral membrane via the light chain and CDRH3, while 4E10 interacts via CDRH1 and CDRH3. 4E10 may also make more extensive interactions with upstream regions of Env (Fig 4E) than 10E8 (Fig 4D) or most likely engage its epitope as fusion intermediate forms after receptor engagement when gp120 regions are no longer in the way. Reduction in the 10E8 interaction with other regions of Env compared with 4E10 may possibly explain its increased neutralization potency.

Our combined design, structural and functional study has provided an explanation for how two extremely broad MPER antibodies engage their common epitopes at the stem of the gp41 ectodomain. The location and conformation of MPER with regard to gp41 and the membrane at different stages of viral fusion remains unclear, but the information presented here helps to fill in missing pieces of the dynamic viral fusion process. These structural and functional insights are important for design of therapeutic antibodies and immunogens as HIV vaccines. The information here can enable the MPER to be linked to lipids in an appropriate orientation as in liposomes or chimeric viruses for vaccination and provide information for improving the pharmacodynamic and pharmacokinetic properties of 10E8 for therapeutic applications.

## Materials and methods

### Protein expression and purification

Genes for 10E8 IgG and Fabs mutants were synthesized by Genscript, Inc. All antibodies and Fabs were expressed in FreeStyle 293S cells (Invitrogen) and purified as described previously [25]. Briefly about 400 μg heavy-chain and 200 μg light-chain vectors were diluted in 25 ml Gibco Opti-MEM I (Invitrogen) reduced-serum medium, sterile filtered, and mixed with 25 ml final volume Opti-MEM I pre-incubated with 1 ml of 293fectin (Invitrogen). After 30 minutes incubation, the mixture was added to 1 L of cells (about 1.2x10^6^ cells/ml density) in FreeStyle 293 expression medium (Invitrogen). The Fabs were purified on a lambda light chain Capture Select affinity column pre-equilibrated with 1x PBS buffer. The unbound material was washed out with the same buffer and the bound Fabs were eluted with 0.1 M glycine, pH 3.0, and immediately neutralized using Tris pH 8.0. Fractions were concentrated and buffer exchanged into 20 mM sodium acetate, pH 5.5, then loaded on a Mono S column (GE Healthcare Life Sciences) equilibrated with the same buffer. The proteins were eluted with a linear gradient of 0 to 50%, 1M KCl in 20 mM sodium acetate, pH 5.6. The concentrated samples were stored in 1xHBS (150 mM NaCl, 10 mM HEPES, pH 7.4).

T117v2 was expressed and purified as previously described [35]. The purified 10E8 variants were incubated in a 1:1 molar ratio with T117v2 scaffold and purified by size exclusion chromatography using a HiLoad 16/600 SuperDex 200pg column (GE Healthcare Life Sciences) in 1xHBS buffer.

### Surface plasmon resonance experiments

Kinetics and affinities of antibody-antigen interactions were measured on a ProteOn XPR36 (Bio-Rad) using GLC Sensor Chip (Bio-Rad) and 1x HBS-EP+ pH 7.4 running buffer (20x stock from Teknova, Cat. No H8022) supplemented with BSA at 1mg/ml. The Human Antibody Capture Kit instructions (Cat. No BR-1008-39 from GE Healthcare Life Sciences) were used to prepare chip surfaces for ligand capture. In a typical experiment, about 6000 RU of capture antibody was amine-coupled in all six flow cells of the GLC Chip. Regeneration was accomplished using 3 M magnesium chloride with 180 seconds contact time and injected four times per each cycle. Raw sensograms were analyzed using ProteOn Manager software (Bio-Rad), including interspot and column double referencing, and either Equilibrium or Kinetic fits with Langmuir model, or both, were employed when applicable. Analyte concentrations were measured on a NanoDrop 2000c Spectrophotometer using Absorption at 280 nm.

### Neutralization

Pseudoviruses were generated by transfection of 293T cells (ATCC) with an HIV-1 Env expressing plasmid and an Env-deficient genomic backbone plasmid (pSG3ΔEnv, (NIH AIDS Reagent Program 11051)), as described previously [51]. Pseudoviruses were harvested 72 hours post-transfection for use in neutralization assays. Neutralizing activity was assessed using a single round of replication pseudovirus assay and TZM-bl target cell (NIH AIDS Reagent Program 8129). TZM-bl cells were seeded at a density of 5,000 cells/well in half-volume white luminescent 96 well plates (Costar 3688), one day prior to assay. Assay and growing medium was Complete DMEM [Dulbecco's Modified Eagle Medium (Corning Cellgro MT15013CV) with 200 μM L-glutamine (Gibco 25030081), 100 U/ml Penicillin-Streptomycin (Invitrogen 15140–122), and ten percent fetal bovine serum (Thermo Scientific HyClone SH3091003)]. To this plate was added pseudovirus, which was preincubated with serial dilutions of antibody for 1 hour at 37°C in duplicate with 25 μl per well final volume. Virus-infected (no serum) and uninfected cell wells were controls on each cell plate. After 24 hours, 75 μl of Complete DMEM was added to each well, bringing the total volume to 100 μl; the plates were replaced in the incubator another 48 hours. Prior to virus signal determination, the liquid medium was removed from the plates, cells were lysed with 45 μl per well Promega Cell Lysis buffer (Product number E1531), and the plates were then shaken for 10 min at 1000 RPM on a Jitterbug Microplate Incubator/Shaker. Thirty μl of Promega Flash substrate (Promega Luciferase 1000 Assay System E4550) was added per well, and luminescence was measured via Synergy 2 Multi-Mode Reader (BioTek).

### Size exclusion chromatography-multi-angle light scattering

SECMALS analysis was performed by separating approximately 50 μg IgG on a Superdex 200 Increase 10/300 GL column (GE Healthcare Life Sciences) in phosphate buffered saline (140 mM NaCl, 2.7 mM KCl, 5.6 mM Na_2_HPO_4_, 1.8 mM KH_2_PO_4_, pH 7.4) and measuring UV absorption at 280 nM and multi-angle light scattering on a DAWN HELEOS II system with Optilab T-rEX refractometer (Wyatt Technology). Raw values were background subtracted and normalized to the maximum signal intensity of each injection. Molecular weights were calculated using the ASTRA6 software package (Wyatt Technology). Data were plotted using GraphPad Prism version 7.0a for Mac (GraphPad Software).

### Protein crystallization

#### Co-crystallization of 10E8-T117v2 complex with lipids

The phosphatidic acid and phosphatidylglycerol lipids were purchased from Avanti Polar Lipids. Each lipid (06:0 PG and 06:0 PA, respectively) solubilized to 15 mM in 20 mM sodium acetate (pH 5.5) was mixed with 10E8/T117v2 complex to obtain a final concentration of 10 mg/ml protein and 8 mM lipid. Crystallization screening was performed with the JCSG/IAVI/Scripps high-throughput CrystalMation robot (Rigaku) at TSRI in a sitting drop vapor diffusion format. The 10E8/T117v2 /06:0 PG crystals grew from a 1:1 (v/v) protein:reservoir solution drop equilibrated against 50% PEG 200, 0.1 M Hepes, pH 7.0, and the 10E8/T117v2 /06:0 PA crystals from a drop equilibrated against 50% PEG 400, 0.2 M NaCl, 0.1 M CHES, pH 9.5. The crystals for each complex were cryo-protected with their respective reservoir solutions.

#### Crystallization of 10E8-T117v2 and 10E8 mutants-T117v2

Small crystals of 10E8-T117v2 complex were obtained in a drop (50%:50% protein:reservoir) equilibrated against 0.1 M Tris pH 7.5, 15% PEG 6,000 reservoir. The crystals were crushed in the reservoir solution and seeded at a the ratio of 16% in a drop (containing 50%: 34% protein:reservoir) equilibrated against 0.1 M Hepes pH 7, 15% PEG 20,000 to produce diffraction-size crystals. The 10E8 mutant-T117v2 complexes concentrated to about 10 mg/ml were also screened for crystallization on our CrystalMation system by sitting drop vapor diffusion. The crystallization conditions and the cryoprotectant solutions used for each complex are reported in S2 Table.

### Data collection, structure determination and refinement

X-ray diffraction data were collected at APS 23ID-B and 23ID-D beam lines and at SSRL on 12–2 and 11–1 beam lines (S1 and S2 Tables) and were auto-indexed and processed with HKL-2000 [52] or XDS [53]. Molecular replacement was performed with Phaser [54] using one of the 10E8 Fabs (PDB 4G6F [25]) and the T117 scaffold (PDB 3LF6 [35]) as search models. Model rebuilding in Coot [55] and refinement with Phenix [56] were performed following an initial rigid body refinement step. The refinement cycles, for structures solved between 2.0 and 2.6 Å, included refinement of individual atomic coordinates, cartesian simulated annealing, refinement of individual isotropic atomic displacement parameters and optimization of X-ray/stereochemistry and X-ray/ADP weights. For the 1.6 Å structure of 10E8 mutant 1 in complex with T117v2, refinement of individual atomic coordinates, cartesian simulated annealing, occupancy and individual atomic displacement parameters refinement with anisotropic ADP for protein atoms and isotropic ADP for solvent were performed as well as optimization of X-ray/stereochemistry and X-ray/ADP weights. X-ray diffraction and refinement statistics are reported in S1 Table for the wild-type 10E8 mature-T117v2 complexes bound to lipids and in S2 Table for 10E8 mutant-T117v2 complexes. Structure figures were generated with Pymol [57]. The atomic coordinates and structure factors of 10E8-T117v2 structures have been deposited in the Protein Data Bank, with the accession codes: 5T6L (for 10E8-T117v2) and 5T85, 5T80 for co-crystals with 06:0 PG and 06:0 PA, respectively and those for 10E8 mutants-T117v2 structures with the accession codes: 5SY8 (for 10E8 mutant 1-T117v2), 5TFW (for 10E8 mutant 2-T117v2), 5T29 (for 10E8 mutant 3-T117v2) and 5T5B (for 10E8 mutant 5-T117v2).

### Viral membrane model using CHARMM

A model of the trimeric MPER epitope-transmembrane region of the gp41 was constructed using PDB 2MOM as a template as described previously [23], with the orientation of the MPER epitope modeled base on crystal structures determined in this study. The structural information on the 10E8 Fab and the PG fragment was transferred to the trimeric model by superposing the MPER epitope region of the T117v2 scaffold to the corresponding region in the model. The crystallographic 06:0 PG lipid fragment was extended to the size of a 1,2-dihexadecanoyl-*sn*-glycero-3-phospho-(1'-*rac*-glycerol) (DPPG) molecule by adding the lipid tails in the direction perpendicular to the plane that includes the light-chain surface residues, the head group of the PG fragment, and Lys683. The putative transmembrane region model constructed solely to anchor the lipid bilayer was then used in membrane building with CHARMM [39]. A rectangular box (x = y = 157.7 Å) was used to generate a heterogeneous bilayer containing 400 lipids on the upper leaflet and 434 lipids on the lower leaflet of the membrane, by replacement method [58]. The HIV-1 membrane lipid composition [47] was taken into account when choosing the composition of the bilayer. The Monte Carlo method was used to place counter potassium ions and NVT (constant volume) ensemble was used during six equilibration steps at a constant temperature of 303 K. The final model has Lys683 of the MPER and the head group of the crystallographically observed lipid embedded into the head group region of the membrane outer leaflet, while the side chains of the residues of the aforementioned light-chain surface are embedded in, or touching, this hydrophilic layer. The model remained stable during equilibration steps.

## Supporting information

S1 Fig Superposition of the 10E8-MPER epitope peptide onto the 10E8-T117v2 structure. The 10E8 Fab-peptide complex (PDB 4G6F; [25]) is colored green. 10E8 Fab in complex with the T117v2 scaffold (gray) is colored brown. The region of the T117v2 scaffold that contains the MPER epitope is highlighted in red.
(PDF)

S2 Fig Electron density maps of ligands bound in the lipid-binding site. (A) Electron density (3σ level; green wires) of a fragment of 06:0 PG (sticks) in the initial difference Fourier map calculated after molecular replacement. (B) 2Fo-Fc density (1σ level; blue wires) for the 06:0 PG fragment (sticks) after the last round of refinement. (C) Electron density (3σ level; green wires) of a fragment of 06:0 PA (sticks) in the initial difference Fourier map calculated after molecular replacement. (D) 2Fo-Fc density (1σ level; blue wires) for the 06:0 PA fragment (sticks) after the last round of refinement. (E) Initial difference Fourier positive electron density (3σ level; green wires) of a glycerol molecule (sticks) bound in the lipid-binding site in the 10E8-T117v2 structure. (F) 2Fo-Fc density (1σ level; blue wires) calculated at final stage of refinement for the glycerol molecule (sticks) bound in the lipid-binding site in the 10E8-T117v2 structure.
(PDF)

S3 Fig SPR sensorgrams of 10E8 epitope-scaffolds. T117v2 binding to 10E8 IgG light-chain mutants.
(PDF)

S4 Fig Structure comparison of 10E8 mutant 5-T117v2 complex to 10E8 wild type-T117v2. (A) Snapshot of the superposition of the light-chain regions of 10E8 in mutant 5 (gray) and wild type (beige) showing regions that deviate slightly from each other. The positions of Cα atoms of each residue are shown as small spheres for comparison. Despite small variations in position of some light-chain residues, the location and conformation of CDRH3 in the two structures (violet, wild type; green, mutant 5) is nearly identical. The region located in the red dashed rectangle is seen in a close-up in (B) for wild type and (C) for mutant 5. The side chains of the residues are shown as sticks, with residues Asn48^(L)^-Phe53^(L)^ adopting multiple conformations. The underlined superscript letters designate the original residues in the 10E8 wild type.
(PDF)

S5 Fig Description of the ligands bound in the lipid-binding site in mutant 1 and 3 structures. (A) Initial Fo-Fc electron density map (3σ level) observed for a glycerol molecule bound in the lipid-binding site in 10E8 mutant 1-T117v2 complex. (B) 2Fo-Fc map (1σ level) for the glycerol in (A) after refinement. (C) All potential glycerol (dark cyan sticks) hydrogen-bond interactions (within ~3.5 Å) with the residues of the lipid-binding site (beige, light chain; violet for heavy chain) in mutant 1 are shown as sticks. (D) Initial Fo-Fc electron density map (3σ level) observed for a phosphate from crystallization conditions bound in the lipid-binding site in 10E8 mutant 3-T117v2 complex. (E) 2Fo-Fc map (1σ level) for the phosphate at (D) after refinement. (F) All potential phosphate (red-orange sticks) hydrogen-bond interactions (within ~3.5 Å) with the residues of the lipid-binding site (beige, light chain; violet, heavy chain) in mutant 3 are shown as sticks. The underlined superscript letters designate the original residues in the 10E8 wild type.
(PDF)

S6 Fig SECMALS analysis of wild type 10E8 and light chain mutants. Normalized UV (red) and light scattering (blue) signals of the indicated antibodies were plotted against the elution volume and protein molar masses of the peaks are indicated in black. Wild-type 10E8 and mutant 1 each show three main peaks in the UV traces, all of which have molecular weights of approximately 150 kDa, indicating that all these peaks represent monomeric IgGs. In contrast, only a single peak can be detected for mutants 2, 3 and 5. Although, scattering signals indicate the presence of high-molecular weight aggregates in the wild type 10E8 and mutant 1 sample, the UV analysis shows that only insignificant amounts (<0.5%) of these aggregates are present in either preparation.
(PDF)

S1 Table X-ray data collection, structure determination and refinement statistics for complexes of 10E8 Fab with T117v2 scaffold alone or co-crystallized with 06:0 PA and 06:0 PG. (PDF)

S2 Table X-ray data collection, structure determination and refinement statistics for complexes of 10E8 Fab mutants with T117v2. (PDF)
