# Supplementary material for: Lipid interactions and angle of approach to the HIV-1 viral membrane of broadly neutralizing antibody 10E8: Insights for vaccine and therapeutic design
Source: PLoS Pathog. 2017 Feb 22;13(2):e1006212. doi: 10.1371/journal.ppat.1006212 (PMC5338832; doi:10.1371/journal.ppat.1006212)

S2 Fig. Electron density maps of ligands bound in the lipid-binding site. (A) Electron density (3σ level; green wires) of a fragment of 06:0 PG (sticks) in the initial difference Fourier map calculated after molecular replacement. (B) 2Fo-Fc density (1σ level; blue wires) for the 06:0 PG fragment (sticks) after the last round of refinement. (C) Electron density (3σ level; green wires) of a fragment of 06:0 PA (sticks) in the initial difference Fourier map calculated after molecular replacement. (D) 2Fo-Fc density (1σ level; blue wires) for the 06:0 PA fragment (sticks) after the last round of refinement. (E) Initial difference Fourier positive electron density (3σ level; green wires) of a glycerol molecule (sticks) bound in the lipid-binding site in the 10E8-T117v2 structure. (F) 2Fo-Fc density (1σ level; blue wires) calculated at final stage of refinement for the glycerol molecule (sticks) bound in the lipid-binding site in the 10E8-T117v2 structure.

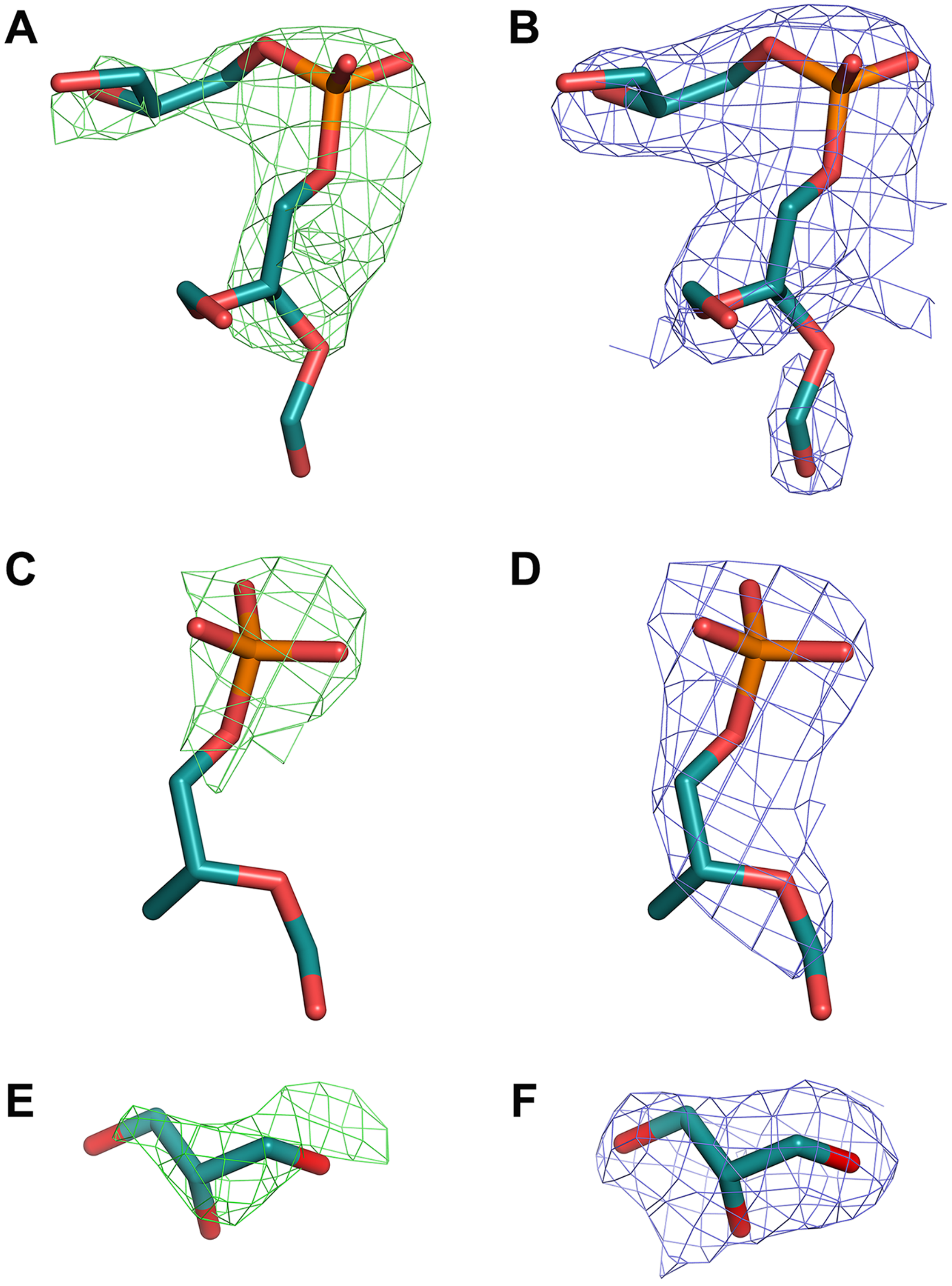

Supplement: S2 Fig — (A) Electron density (3σ level; green wires) of a fragment of 06:0 PG (sticks) in the initial difference Fourier map calculated after molecular replacement. (B) 2Fo-Fc density (1σ level; blue wires) for the 06:0 PG fragment (sticks) after the last round of refinement. (C) Electron density (3σ level; green wires) of a fragment of 06:0 PA (sticks) in the initial difference Fourier map calculated after molecular replacement. (D) 2Fo-Fc density (1σ level; blue wires) for the 06:0 PA fragment (sticks) after the last round of refinement. (E) Initial difference Fourier positive electron density (3σ level; green wires) of a glycerol molecule (sticks) bound in the lipid-binding site in the 10E8-T117v2 structure. (F) 2Fo-Fc density (1σ level; blue wires) calculated at final stage of refinement for the glycerol molecule (sticks) bound in the lipid-binding site in the 10E8-T117v2 structure. (PDF) [file ppat.1006212.s002.pdf]
