# Supplementary material for: Lipid interactions and angle of approach to the HIV-1 viral membrane of broadly neutralizing antibody 10E8: Insights for vaccine and therapeutic design
Source: PLoS Pathog. 2017 Feb 22;13(2):e1006212. doi: 10.1371/journal.ppat.1006212 (PMC5338832; doi:10.1371/journal.ppat.1006212)

S3 Fig. SPR sensorgrams of 10E8 epitope-scaffolds. T117v2 binding to 10E8 IgG light-chain mutants.

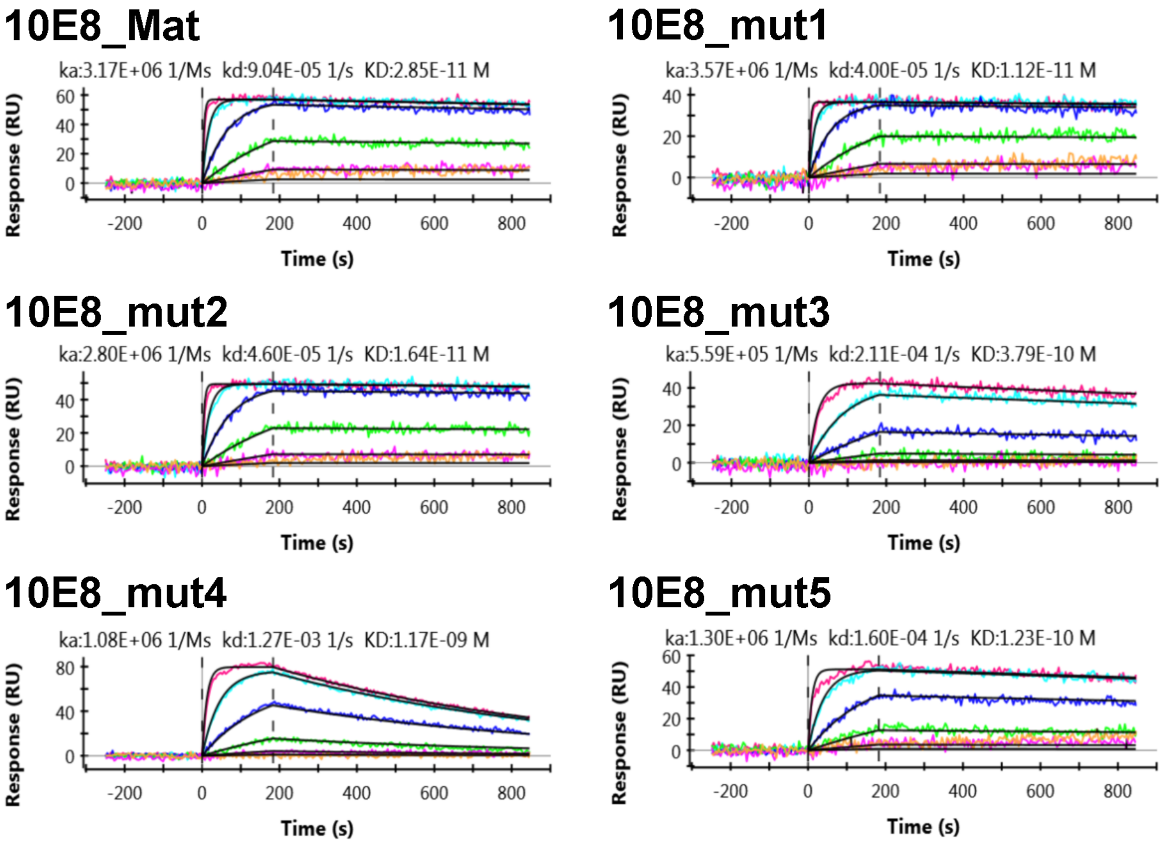

Supplement: S3 Fig — T117v2 binding to 10E8 IgG light-chain mutants. (PDF) [file ppat.1006212.s003.pdf]
