# Supplementary material for: Lipid interactions and angle of approach to the HIV-1 viral membrane of broadly neutralizing antibody 10E8: Insights for vaccine and therapeutic design
Source: PLoS Pathog. 2017 Feb 22;13(2):e1006212. doi: 10.1371/journal.ppat.1006212 (PMC5338832; doi:10.1371/journal.ppat.1006212)

S4 Fig. Structure comparison of 10E8 mutant 5-T117v2 complex to 10E8 wild type-T117v2. (A) Snapshot of the superposition of the light-chain regions of 10E8 in mutant 5 (gray) and wild type (beige) showing regions that deviate slightly from each other. The positions of Cα atoms of each residue are shown as small spheres for comparison. Despite small variations in position of some light-chain residues, the location and conformation of CDRH3 in the two structures (violet, wild type; green, mutant 5) is nearly identical. The region located in the red dashed rectangle is seen in a close-up in (B) for wild type and (C) for mutant 5. The side chains of the residues are shown as sticks, with residues Asn48$^{(L)}$-Phe53$^{(L)}$ adopting multiple conformations. The underlined superscript letters designate the original residues in the 10E8 wild type.

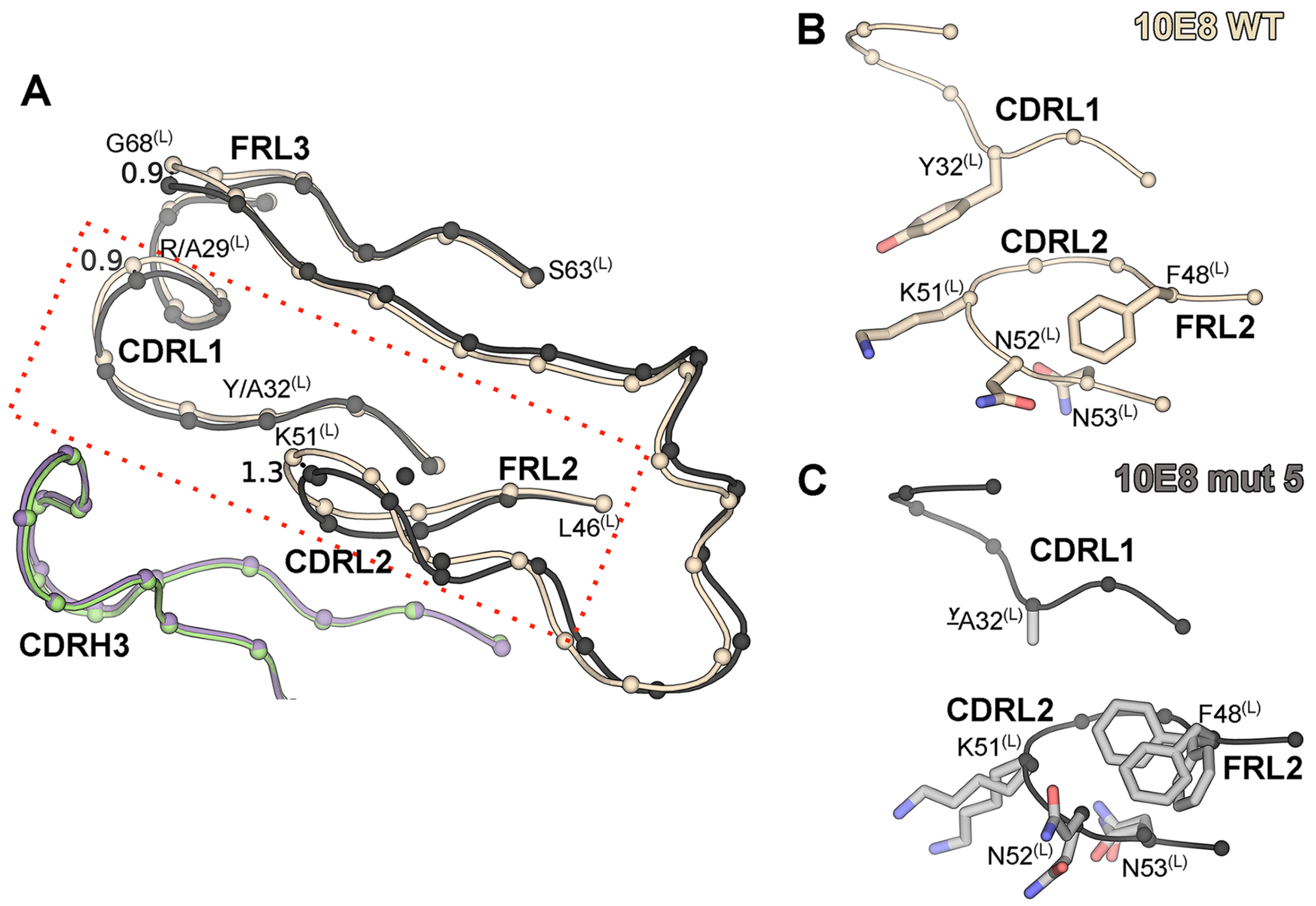

Supplement: S4 Fig — (A) Snapshot of the superposition of the light-chain regions of 10E8 in mutant 5 (gray) and wild type (beige) showing regions that deviate slightly from each other. The positions of Cα atoms of each residue are shown as small spheres for comparison. Despite small variations in position of some light-chain residues, the location and conformation of CDRH3 in the two structures (violet, wild type; green, mutant 5) is nearly identical. The region located in the red dashed rectangle is seen in a close-up in (B) for wild type and (C) for mutant 5. The side chains of the residues are shown as sticks, with residues Asn48(L)-Phe53(L) adopting multiple conformations. The underlined superscript letters designate the original residues in the 10E8 wild type. (PDF) [file ppat.1006212.s004.pdf]
