# Supplementary material for: Lipid interactions and angle of approach to the HIV-1 viral membrane of broadly neutralizing antibody 10E8: Insights for vaccine and therapeutic design
Source: PLoS Pathog. 2017 Feb 22;13(2):e1006212. doi: 10.1371/journal.ppat.1006212 (PMC5338832; doi:10.1371/journal.ppat.1006212)

S1 Fig. Superposition of the 10E8-MPER epitope peptide onto the 10E8-T117v2 structure. The 10E8 Fab-peptide complex (PDB 4G6F; [25]) is colored green. 10E8 Fab in complex with the T117v2 scaffold (gray) is colored brown. The region of the T117v2 scaffold that contains the MPER epitope is highlighted in red.

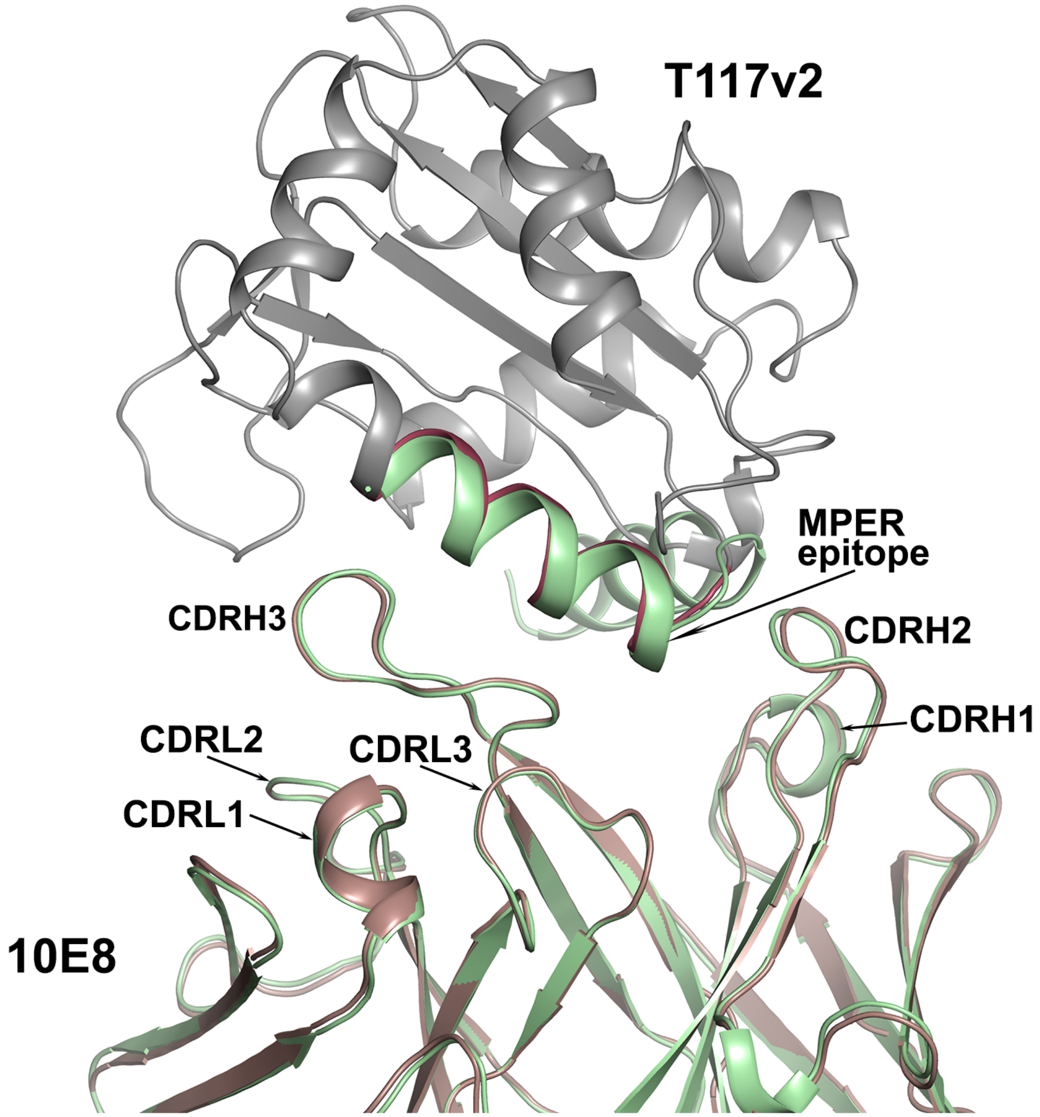

Supplement: S1 Fig — The 10E8 Fab-peptide complex (PDB 4G6F; [25]) is colored green. 10E8 Fab in complex with the T117v2 scaffold (gray) is colored brown. The region of the T117v2 scaffold that contains the MPER epitope is highlighted in red. (PDF) [file ppat.1006212.s001.pdf]
