# Supplementary material for: Lipid interactions and angle of approach to the HIV-1 viral membrane of broadly neutralizing antibody 10E8: Insights for vaccine and therapeutic design
Source: PLoS Pathog. 2017 Feb 22;13(2):e1006212. doi: 10.1371/journal.ppat.1006212 (PMC5338832; doi:10.1371/journal.ppat.1006212)

S6 Fig. SECMALS analysis of wild type 10E8 and light chain mutants. Normalized UV (red) and light scattering (blue) signals of the indicated antibodies were plotted against the elution volume and protein molar masses of the peaks are indicated in black. Wild-type 10E8 and mutant 1 each show three main peaks in the UV traces, all of which have molecular weights of approximately 150 kDa, indicating that all these peaks represent monomeric IgGs. In contrast, only a single peak can be detected for mutants 2, 3 and 5. Although, scattering signals indicate the presence of high-molecular weight aggregates in the wild type 10E8 and mutant 1 sample, the UV analysis shows that only insignificant amounts (<0.5%) of these aggregates are present in either preparation.

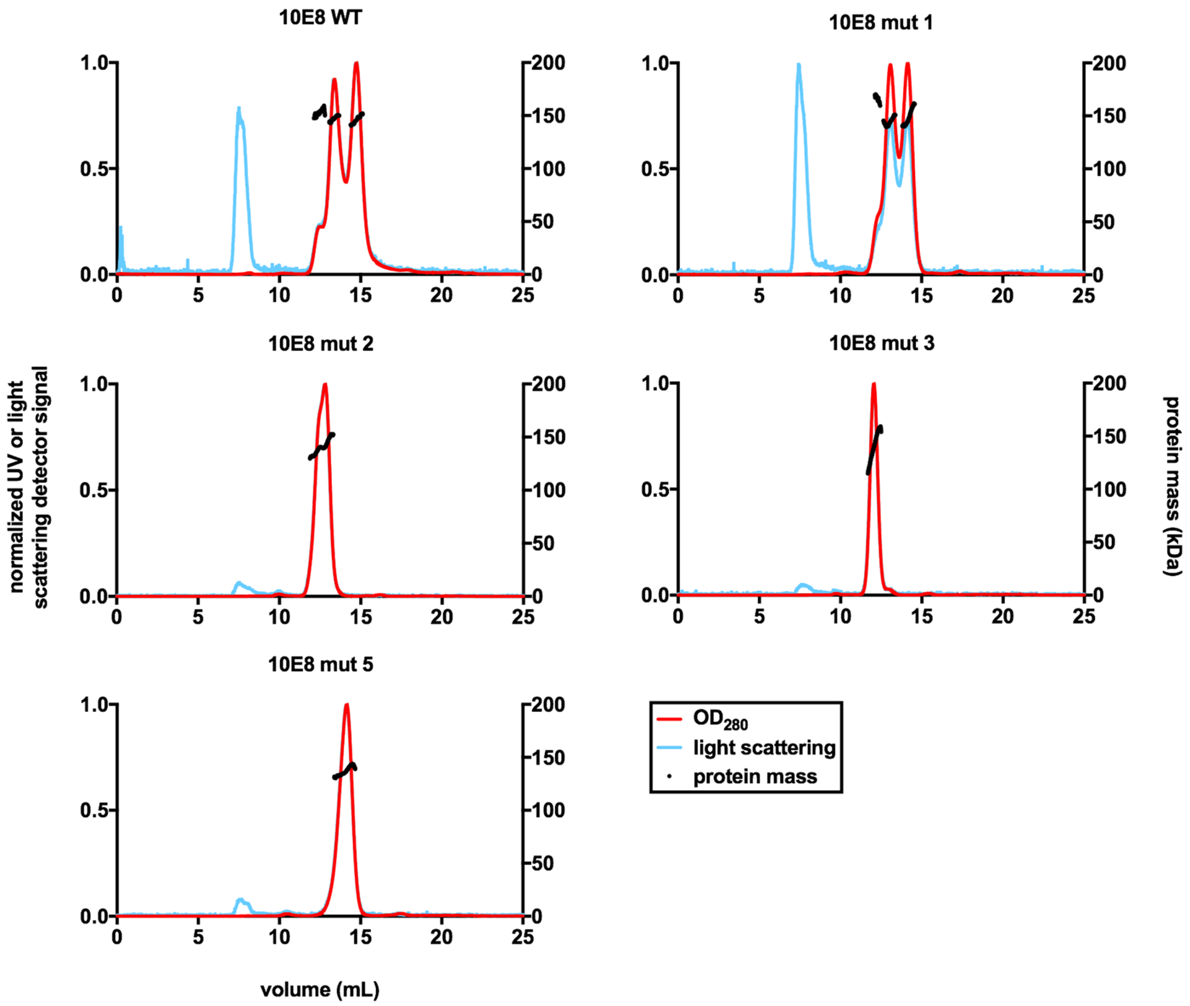

Supplement: S6 Fig — Normalized UV (red) and light scattering (blue) signals of the indicated antibodies were plotted against the elution volume and protein molar masses of the peaks are indicated in black. Wild-type 10E8 and mutant 1 each show three main peaks in the UV traces, all of which have molecular weights of approximately 150 kDa, indicating that all these peaks represent monomeric IgGs. In contrast, only a single peak can be detected for mutants 2, 3 and 5. Although, scattering signals indicate the presence of high-molecular weight aggregates in the wild type 10E8 and mutant 1 sample, the UV analysis shows that only insignificant amounts (<0.5%) of these aggregates are present in either preparation. (PDF) [file ppat.1006212.s006.pdf]
