# Supplementary material for: Lipid interactions and angle of approach to the HIV-1 viral membrane of broadly neutralizing antibody 10E8: Insights for vaccine and therapeutic design
Source: PLoS Pathog. 2017 Feb 22;13(2):e1006212. doi: 10.1371/journal.ppat.1006212 (PMC5338832; doi:10.1371/journal.ppat.1006212)
Supplement: S1 Table — (PDF) [file ppat.1006212.s007.pdf]

S1 Table. X-ray data collection, structure determination and refinement statistics for complexes of 10E8 Fab with T117v2 scaffold alone or co-crystallized with 06:0 PA and 06:0 PG

**Complex**	**10E8 Fab -T117v2-06:0 PG**	**10E8 Fab -T117v2-06:0 PA**	**10E8 Fab -T117v2**
**Reservoir condition**	50% PEG 200, 0.1 M Hepes pH 7.0	50% PEG 400, 0.2M NaCl, 0.1 M CHES pH 9.5	0.1 M Hepes pH 7, 15% PEG 20,000
**Cryoprotectant**	as above	as above	+ 26% glycerol
**Data collection**			
X-ray source	SSRL	SSRL	APS
Beam-line	11-1	12-2	23ID-B
Detector	Pilatus	Pilatus	MARMosaic 300
Wavelength (Å)	0.97945	0.97950	1.03314
Temperature (K)	110	110	110
No. crystals	1	1	1
Space group	$P2_12_12_1$	$P2_12_12_1$	$P2_1$
Unit cell (a, b, and c; Å)<br>(α, β, and γ; °)	55.00, 67.28,194.67<br>90.0, 90.0, 90.0	55.22, 67.26, 196.84<br>90.0, 90.0, 90.0	41.10, 158.97, 99.69<br>90.0, 97.98, 90.0
Resolution range (Å)	48.67-2.37	46.96-2.62	47.14- 2.10
Highest resolution shell[a]	2.40-2.37	2.69-2.62	2.15-2.10
No. measurements	100,173 (5,406)	85,286 (4,009)	171,753 (11,873)
No. unique reflections	28,788 (1,461)	21,918 (1,055)	72,210 (5,086)
Redundancy	3.5 (3.7)	3.9 (3.8)	2.4 (2.3)
Completeness (%)	95.7 (98.1)	97.5 (96.7)	97.5 (91.9)
R_{sym}[b]	8.6 (70.2)	7.5 (66.6)	7.4 (63.0)
R_{pim}[c]	5.2 (40.6)	4.3 (37.4)	5.8 (48.1)
$CC_{1/2}$[d]	91.5 (62.7)	96.0 (82.2)	99.7 (74.5)
Signal to noise ($\langle I/\sigma I \rangle$)	8.5 (1.8)	9.8 (2.1)	9.3 (1.6)
Solvent content (%)	55.4	56.4	51.0
**Refinement**			
No. reflections used for refinement	28,737	21,841	72,183
R_{cryst}[d] (%)	19.5	18.4	17.8
R_{free}[e] (%)	25.0	24.3	21.8
**Model composition (asymmetric unit)**			
10E8 Fab	1	1	2
T117v2	1	1	2
06:0 PG	1	n/a	n/a
06:0 PA	n/a	1	n/a
PEG	2	-	-
Glycerol	n/a	n/a	17
HEPES	-	-	1
Waters	147	61	405
**B-values**			
Wilson plot (Å^2)	62	75	34
Mean isotropic (Å^2)	70	87	53
Mean isotropic protein/solvent (Å^2)	70 / 69	86 / 81	53 / 50
Mean isotropic ligand<br>in lipid-binding site (Å^2)	106 (06:0 PG)	134 (06:0 PA)	59 (glycerol)
**R.m.s.d. from ideal values (Å)**			
Bond lengths (Å)	0.008	0.009	0.008
Bond angles (°)	1.10	1.09	0.88
**Ramachandran**			
Most favored regions (%	95.5	96.7	95.6
Additional allowed regions (%)	3.6	2.4	3.4
Disallowed regions (%)	0.9	0.9	1.0

[a] Values in parentheses correspond to the highest resolution shells

[b] $R_{sym} = \Sigma_{hkl} \Sigma_{j = 1,n} | \langle I_{hkl} \rangle - I_{hklj}| / \Sigma_{hkl} \Sigma_{j = 1,n} |I_{hklj}|$, where the outer sum (hkl) is taken over the unique reflections

[c] $R_{pim} = \Sigma_{hkl}[1/(n-1)]^{1/2} \Sigma_{i=1,n}|I_{hkli} - <I_{hkl}>| / \Sigma_{hkl} \Sigma_{i = 1,n}|I_{hkli}|$

[d] $CC_{1/2}$ = Pearson Correlation Coefficient between two random half datasets

[e] $R_{cryst} = \Sigma_{hkl}||F_{o,hkl}| - k|F_{c,hkl}||/\Sigma_{hkl}|F_{o,hkl}|$, where $|F_{o,hkl}|$ and $|F_{c,hkl}|$ are the observed and calculated structure factor amplitudes, respectively

[f] R_{free} , as for R_{cryst}, but for a set of reflections (5% of the total) omitted from refinement

n/a, not applicable
