# Supplementary material for: Lipid interactions and angle of approach to the HIV-1 viral membrane of broadly neutralizing antibody 10E8: Insights for vaccine and therapeutic design
Source: PLoS Pathog. 2017 Feb 22;13(2):e1006212. doi: 10.1371/journal.ppat.1006212 (PMC5338832; doi:10.1371/journal.ppat.1006212)
Supplement: S2 Table — (PDF) [file ppat.1006212.s008.pdf]

S2 Table. X-ray data collection, structure determination and refinement statistics for complexes of 10E8 Fab mutants with T117v2

**Complex**	**10E8 Fab mutant 1-T117v2**	**10E8 Fab mutant 2-T117v2**	**10E8 Fab mutant 3-T117v2**	**10E8 Fab mutant 5-T117v2**
**Reservoir condition**	0.1 M Hepes pH 7.5, 20% PEG 8000	0.2 M Na thiocyanate, 20% PEG 3350, pH 6.9, 10% ethylene glycol	0.1 M phosphate-citrate pH 4.2, 40% PEG 600	0.1 M Hepes pH 7.0, 30% PEG 6000
**Cryoprotectant**	+26% glycerol	+26% ethylene glycol	as above	+26% glycerol
**Data collection**				
X-ray source	SSRL	APS	SSRL	APS
Beam-line	12-2	23ID-B	12-2	23ID-D
Detector	Pilatus	MARMosaic-300	Pilatus	Pilatus
Wavelength (Å)	0.97950	1.03320	0.97950	1.03320
Temperature (K)	110	110	110	110
No. of crystals	1	1	1	1
Space group	P1	C2	C2	$P2_1$
Unit cell (a, b, and c; Å)<br>(α, β, and γ; °)	41.40, 56.59, 64.20<br>101.75, 101.19, 92.16	213.49, 63.41, 53.87<br>90.0, 95.79, 90.0	110.88, 66.97, 84.26<br>90.0, 101.46, 90.0	40.93, 159.00,100.61<br>90.0, 95.17, 90.0
Resolution range (Å)	40.49-1.62	47.24-2.17	36.56-2.03	37.88-2.07
Highest resolution shell[a]	1.65-1.62	2.17-2.22	2.07-2.03	2.11-2.07
No. measurements	214,487 (8,100)	151,215 (10,717)	89,393 (3,001)	158,258 (6,770)
No. unique reflections	61,518 (2,893)	36,138 (2,687)	36,193 (1,667)	73,937 (3,563)
Redundancy	3.5 (2.8)	4.2 (4.0)	2.5 (1.8)	2.1 (1.9)
Completeness (%)	86.9 (82.0)	94.5 (95.8)	92.1 (87.0)	94.8 (91.4)
R_{sym}[b]	8.7 (71.1)	5.6 (63.5)	8.1 (53.5)	4.9 (45.6)
R_{pim}[c]	5.3 (45.5)	3.1 (35.6)	5.8 (45.9)	3.9 (38.3)
$CC_{1/2}$[d]	94.3 (75.3)	99.9 (83.5)	86.1 (58.0)	95.0 (68.0)
Signal to noise ($\langle I/\sigma I \rangle$)	9.01 (2.4)	15.50 (2.1)	7.6 (2.5)	9.2 (2.1)
Solvent content (%)	43.9	55.8	49.3	54.4
**Refinement**				
No. reflections used for refinement	61,470	36,126	36,187	73,905
R_{cryst}[e] (%)	17.2	16.9	17.4	17.8
R_{free}[f] (%)	21.4	22.8	21.3	21.6
**Model composition (asymmetric unit)**				
10E8 Fab	1	1	1	2
T117v2	1	1	1	2
Glycerol	4	n/a	n/a	7
Ethylene glycol	n/a	1	n/a	n/a
Phosphate	n/a	n/a	1	n/a
PEG	-	1	1	-
Waters	351	233	144	209
**B-values**				
Wilson plot (Å^2)	22	41	37	48
Mean isotropic (Å^2)	31	59	54	70
Mean isotropic protein/solvent (Å^2)	30 / 41	60 / 54	54 / 51	71 / 60
Mean isotropic ligand in lipid-binding site (Å^2)	56 (glycerol)	43 (water)	88 (phosphate)	86 (water)
**R.m.s.d. from ideal values (Å)**				
Bond lengths (Å)	0.006	0.007	0.007	0.008
Bond angles (°)	1.03	0.87	1.01	0.87
**Ramachandran**				
Most favored regions (%)	97.1	95.8	96.3	96.7
Additional allowed regions (%)	2.5	3.3	3.0	2.5
Disallowed regions (%)	0.4	0.9	0.7	0.8

[a] Values in parentheses correspond to the highest resolution shells

[b] $R_{sym} = \Sigma_{hkl} \Sigma_{j=1,N} | \langle I_{hkl} \rangle - I_{hklj}| / \Sigma_{hkl} \Sigma_{j=1,N} |I_{hklj}|$, where the outer sum (hkl) is taken over the unique reflections

[c] $R_{pim} = \Sigma_{hkl}[1/(N-1)]^{1/2} \Sigma_{i=1,N}|I_{hkli} - \langle I_{hkl} \rangle| / \Sigma_{hkl} \Sigma_{i=1,N}|I_{hkli}|$

[d] $CC_{1/2}$ = Pearson Correlation Coefficient between two random half datasets

[e] $R_{cryst} = \Sigma_{hkl}||F_{o,hkl}| - k|F_{c,hkl}||/\Sigma_{hkl}|F_{o,hkl}|$, where $|F_{o,hkl}|$ and $|F_{c,hkl}|$ are the observed and calculated structure factor amplitudes, respectively

[f] R_{free} , as for R_{cryst}, but for a set of reflections (5% of total) omitted from refinement

n/a, not applicable
