# Supplementary material for: Lipid interactions and angle of approach to the HIV-1 viral membrane of broadly neutralizing antibody 10E8: Insights for vaccine and therapeutic design
Source: PLoS Pathog. 2017 Feb 22;13(2):e1006212. doi: 10.1371/journal.ppat.1006212 (PMC5338832; doi:10.1371/journal.ppat.1006212)

S5 Fig. Description of the ligands bound in the lipid-binding site in mutant 1 and 3 structures. (A) Initial Fo-Fc electron density map (3σ level) observed for a glycerol molecule bound in the lipid-binding site in 10E8 mutant 1-T117v2 complex. (B) 2Fo-Fc map (1σ level) for the glycerol in (A) after refinement. (C) All potential glycerol (dark cyan sticks) hydrogen-bond interactions (within ~3.5 Å) with the residues of the lipid-binding site (beige, light chain; violet for heavy chain) in mutant 1 are shown as sticks. (D) Initial Fo-Fc electron density map (3σ level) observed for a phosphate from crystallization conditions bound in the lipid-binding site in 10E8 mutant 3-T117v2 complex. (E) 2Fo-Fc map (1σ level) for the phosphate at (D) after refinement. (F) All potential phosphate (red-orange sticks) hydrogen-bond interactions (within ~3.5 Å) with the residues of the lipid-binding site (beige, light chain; violet, heavy chain) in mutant 3 are shown as sticks. The underlined superscript letters designate the original residues in the 10E8 wild type.

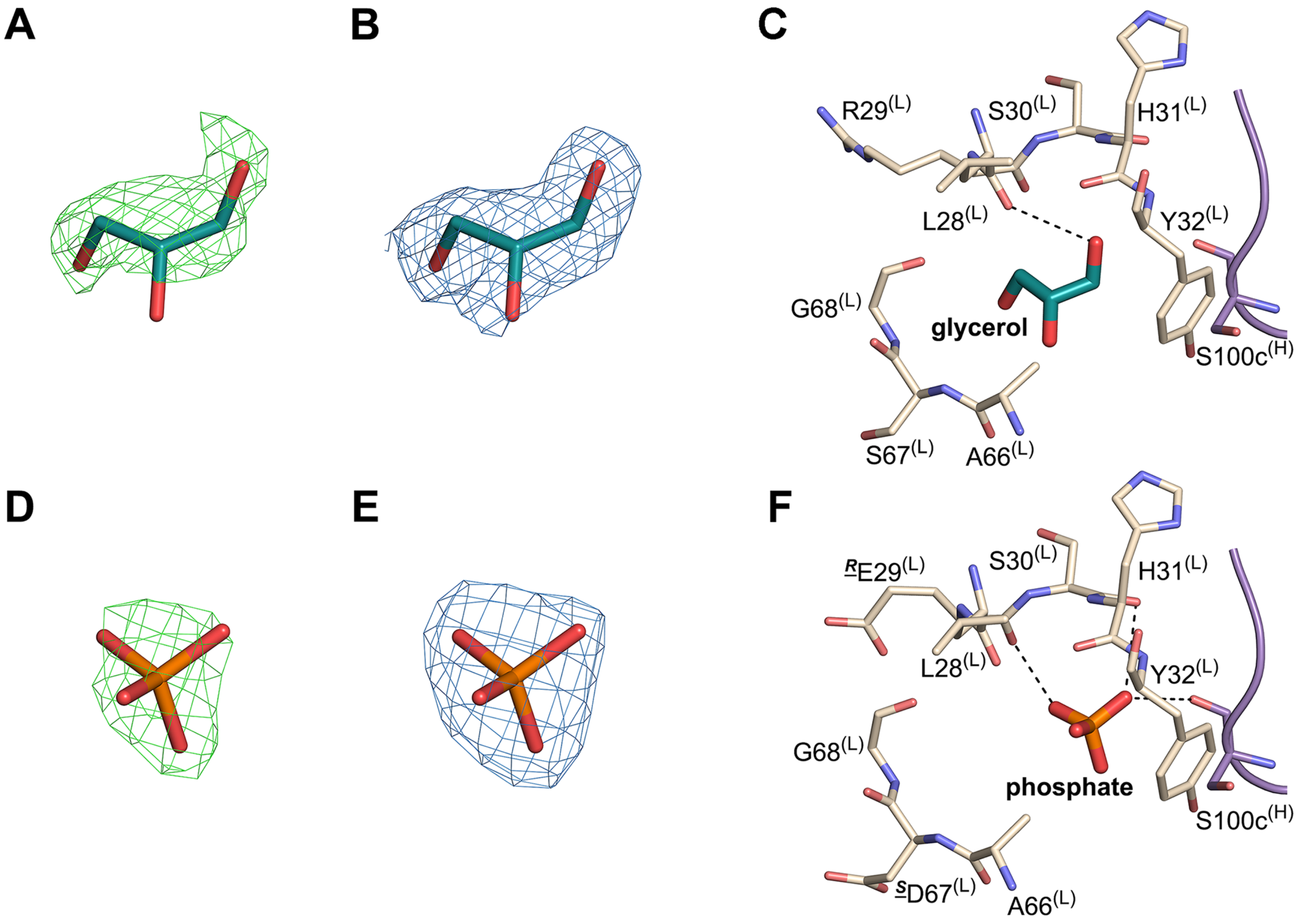

Supplement: S5 Fig — (A) Initial Fo-Fc electron density map (3σ level) observed for a glycerol molecule bound in the lipid-binding site in 10E8 mutant 1-T117v2 complex. (B) 2Fo-Fc map (1σ level) for the glycerol in (A) after refinement. (C) All potential glycerol (dark cyan sticks) hydrogen-bond interactions (within ~3.5 Å) with the residues of the lipid-binding site (beige, light chain; violet for heavy chain) in mutant 1 are shown as sticks. (D) Initial Fo-Fc electron density map (3σ level) observed for a phosphate from crystallization conditions bound in the lipid-binding site in 10E8 mutant 3-T117v2 complex. (E) 2Fo-Fc map (1σ level) for the phosphate at (D) after refinement. (F) All potential phosphate (red-orange sticks) hydrogen-bond interactions (within ~3.5 Å) with the residues of the lipid-binding site (beige, light chain; violet, heavy chain) in mutant 3 are shown as sticks. The underlined superscript letters designate the original residues in the 10E8 wild type. (PDF) [file ppat.1006212.s005.pdf]
